# Bilateral *en-masse* distalization of maxillary posterior teeth with skeletal anchorage: a case report

**DOI:** 10.1590/2177-6709.21.3.085-093.oar

**Published:** 2016

**Authors:** Saeed Noorollahian, Shiva Alavi, Farinaz Shirban

**Affiliations:** 1 Assistant Professor, Isfahan University of Medical Sciences, Dental Implants Research Center, Department of Orthodontics, School of Dentistry, Isfahan, Iran.; 2 Associate Professor, Isfahan University of Medical Sciences, Dental Materials Research Center, Department of Orthodontics, School of Dentistry, Isfahan, Iran.; 3 Assistant Professor, Isfahan University of Medical Sciences, Torabinejad Dental Research Center, Department of Orthodontics, School of Dentistry, Isfahan, Iran.

**Keywords:** Hyrax, Orthodontics, Tooth movement

## Abstract

**Objective:**

The aim of this study was to introduce a new method for bilateral distal movement of the entire maxillary posterior segment.

**Case report:**

A 17-year-old girl with Class I skeletal malocclusion (end-to-end molar relationships, deviated midline and space deficiency for left maxillary canine) was referred for orthodontic treatment. She did not accept maxillary first premolars extraction. A modified Hyrax appliance (Dentaurum Ispringen, Germany) was used for bilateral distalization of maxillary posterior teeth simultaneously. Expansion vector was set anteroposteriorly. Posterior legs of Hyrax were welded to first maxillary molar bands. All posterior teeth on each side consolidated with a segment of 0.017 × 0.025-in stainless steel wire from the buccal side. Anterior legs of Hyrax were bent into eyelet form and attached to the anterior palate with two mini-screws (2 × 10 mm) (Jeil Medical Corporation Seoul, South Korea). Hyrax opening rate was 0.8 mm per month. Lateral cephalometric radiographs were used to evaluate the extent of distal movement. 3.5-mm distalization of posterior maxillary teeth was achieved in five months.

**Results:**

A nearly bodily distal movement without anchorage loss was obtained.

**Conclusion:**

The mini-screw-supported modified Hyrax appliance was found to be helpful for achieving *en-masse* distal movement of maxillary posterior teeth.

## RESUMO

**Objetivo:**

o objetivo do presente estudo foi apresentar um novo método para a distalização bilateral de todo o segmento posterior superior.

**Relato do caso:**

uma jovem de 17 anos de idade, com má oclusão esquelética de Classe I (relação de molares em topo a topo, linha média desviada e deficiência de espaço para o canino superior esquerdo) foi encaminhada para tratamento ortodôntico, mas recusou-se a autorizar a extração dos primeiros pré-molares superiores. Um aparelho Hyrax modificado (Dentaurum, Ispringen, Alemanha) foi utilizado para a distalização bilateral simultânea dos dentes posteriores superiores. O vetor de expansão foi definido no sentido anteroposterior. As barras de conexão posteriores do Hyrax foram soldadas às bandas dos primeiros molares superiores. Em cada um dos lados, todos os dentes posteriores foram agrupados com um segmento de fio de aço inoxidável de calibre 0,017" x 0,025" passando por vestibular. As barras de conexão anteriores do Hyrax foram dobradas em forma de *loop* e conectadas à região anterior do palato por meio de dois mini-implantes (2 x 10mm) (Jeil Medical Corporation, Seoul, Coreia do Sul). A taxa de abertura do Hyrax foi de 0,8mm / mês. Telerradiografias laterias foram usadas para avaliar a extensão dos movimentos de distalização.

**Resultados:**

em um prazo de cinco meses, foi obtida uma distalização de 3,5mm dos dentes posteriores superiores. O movimento realizado foi praticamente de corpo (translação), sem qualquer perda de ancoragem.

**Conclusão:**

o aparelho Hyrax modificado suportado por mini-implantes mostrou-se útil para efetuar o movimento de distalização em massa dos dentes posteriores superiores.

## INTRODUCTION

Arch-length deficiency is a common problem in Orthodontics. We have two choices to manage this discrepancy: arch expansion or tooth mass reduction.^1^ When space deficiency is combined with missing or previous extracted teeth and a tendency towards molar Class II relationship, the first choice for providing space and solve the problem is distal movement of posterior teeth. This option is also recommended for patients who have space deficiency, but refuse tooth extraction.

Traditional techniques for molar distalization are extra-oral traction,^2^ ^,^ ^3^Cetlin removable plate,^4^ ^,^ ^5^ Wilson arches^6^ and First Class Appliance (Leone, Firenze, Italy) with continuous force delivered by springs, which counterbalances the action of buccal screws.^7^ ^,^ ^8^ All these distalizing appliances rely partially or totally on patient's cooperation.

Different sources of force were used for distal driving: repelling magnets,^9^ ^,^ ^10^ coil springs, looped NiTi wires,^11^ super-elastic nickel-titanium arch wires,^12^ coil springs on a sectional arch wire (Jones Jig assembly,^13^ ^,^ ^14^ ^,^ ^15^distal jet^16^ ^-^ ^18^ and Keles slider^19^) springs in beta titanium alloy (pendulum appliance,^15^ ^,^ ^20^ ^,^ ^21^ K-loop^22^, Intraoral Bodily Molar Distalizer Pendulum (IBMB),^23^ expansion screws (Modified Pendulum Appliance^24^ and Frog Appliance^25^).

Routine anchorage units used in these appliances are other teeth or palatal acrylic pad.^26^ Recently, bone-borne appliances, such as dental implants,^27^ fixation mini-plates^28^ and orthodontic mini-screws^29^ ^-^ ^33^ have become widely used as anchorage system; for instance, Graz implant-supported pendulum appliance,^28^ bone-anchored pendulum appliance,^29^ ^-^ ^32^ a mini-screw implant-supported distalization system (MISDS),^33^ the ZGA (Zygoma-Gear Appliance) anchorage system for buccal segment distalization,^34^ ^,^ ^35^ ^,^ ^36^ dual-force distalizer supported by mini-implants (DFD),^37^ mesialy extended TPA (ME-TPA) with skeletal anchorage,^38^ the Keles Slider appliance with a palatal implant^39^ and timely relocation of mini-implants for uninterrupted full-arch distalization (jig).^40^

The aim of this report was to introduce a new method for simultaneous bilateral distalalization of the entire maxillary posterior segment.

## DIAGNOSIS

**Figure 1:**
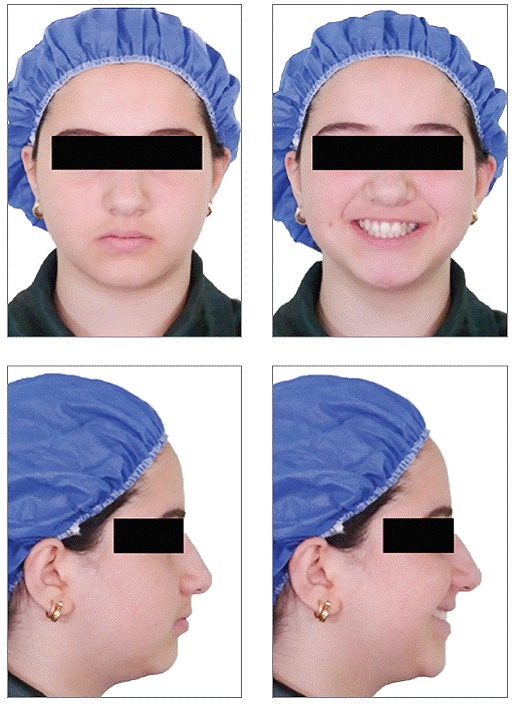
Pretreatment extraoral photographs.

**Figure 2:**
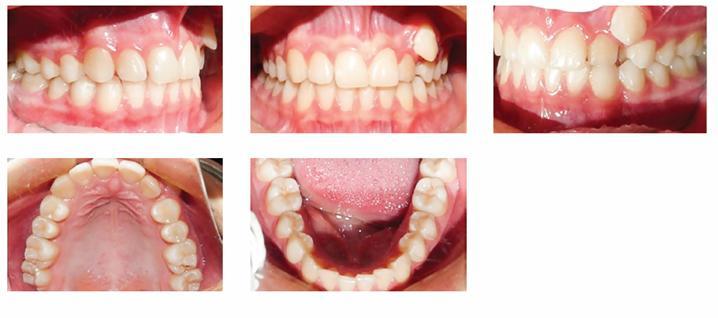
Pretreatment intraoral photographs.

A 17-year-old female patient visited the orthodontic department of Isfahan University of Medical Sciences. Her chief complaint was malposition of anterior teeth. She did not have any medical problems or active periodontal disease. The patient had a symmetrical, mesoprosopic and balanced face and a mild convex profile.(Fig 1). Intraoral examination revealed buccally displaced maxillary left canine, 3.5-mm upper midline deviation to the left and end-to-end molar relationship (Fig 2). Cephalometric analysis revealed no skeletal discrepancy.

## TREATMENT ALTERNATIVES

Two treatment options were proposed to the patient: 

 1) Extraction of maxillary first premolars.

 2) Distalization of the entire posterior segment.

The patient preferred the second treatment option.

## TREATMENT PROGRESS

**Figure 3:**
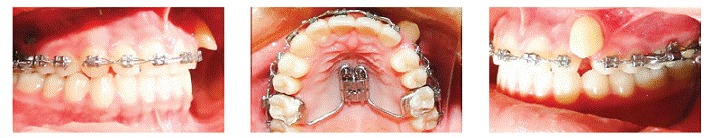
Pre-distal driving intraoral photographs.

Treatment process began after extraction of maxillary third molars. A modified Hyrax appliance (Dentaurum Ispringen, Germany) was used for bilateral distalization of maxillary posterior teeth, simultaneously. The expansion vector was set anteroposteriorly. Posterior legs of Hyrax were welded to first maxillary molar bands. All posterior teeth on each side consolidated with a segment of 0.017 × 0.025-in stainless steel wire from the buccal side. Anterior legs of Hyrax were bent into eyelet form and attached to the anterior palate with two mini-screws (2 × 10 mm) (Jeil Medical Corporation, Seoul, South Korea) (Fig 3). Hyrax opening rate was 0.8 mm per month. Lateral cephalometric radiographs were used to evaluate the extent of distal movements.

**Figure 4:**
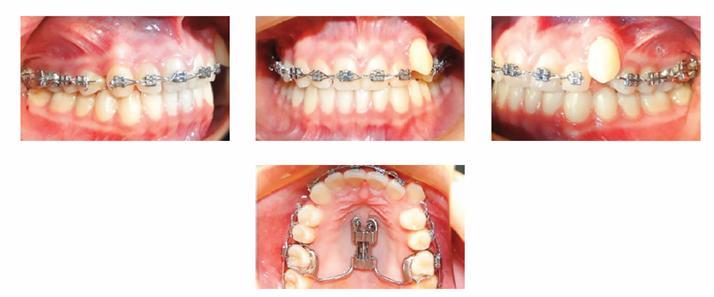
Intraoral photographs after distal driving completion.

The stability of the appliance, mini-screws and oral hygiene were evaluated at each one of the monthly appointments. After five months, Class I relationship in molars and premolars was obtained. Post-distal driving intraoral view is seen in Figure 4. Cephalometric analysis was carried out to assess changes of molar position, inclination, mandibular plane angle and mini-screw inclination alternations. 

## TREATMENT RESULTS

**Figure 5:**
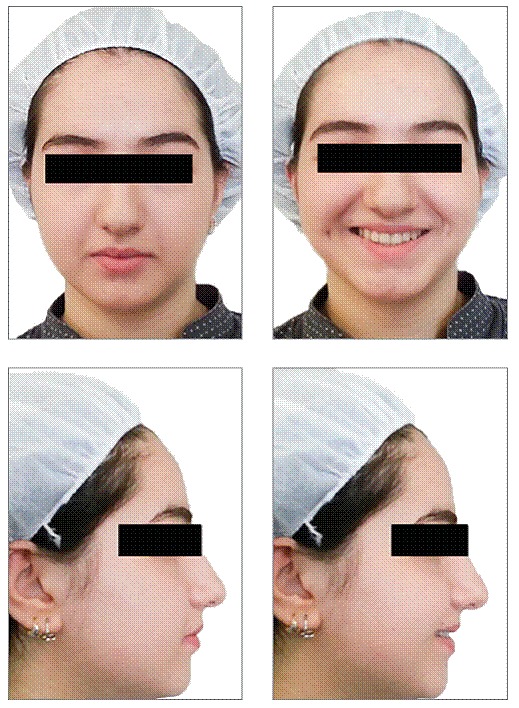
Post-treatment extraoral photographs.

Figures 5 and ^6^ show the final outcomes after 15 months of orthodontic therapy. Buccally displaced maxillary left canine was corrected by using the space resulting from distal driving on the left side and midline correction by using the space resulting from distal driving on the right side. Molar and canine relationship was corrected, Class I was achieved and midline improved.

**Figure 6:**
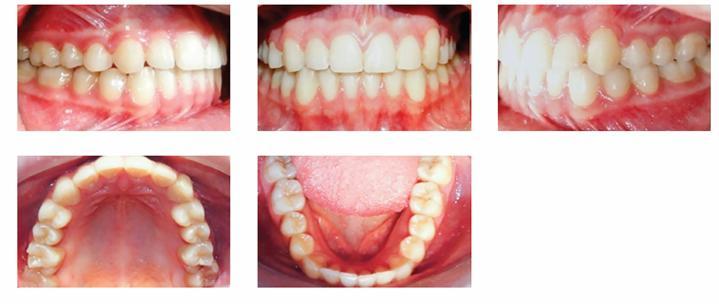
Post-treatment intraoral photographs.

**Figure 7:**
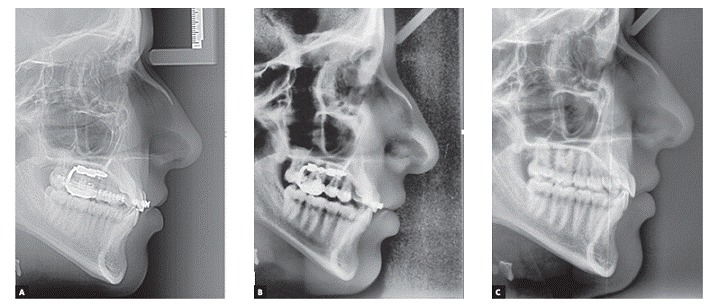
A) Pre-distal driving lateral cephalometry. B) Post-distal driving lateral cephalometry. C) Post-treatment lateral cephalometry.

Figures 7 and ^8^ show pre-distal driving, post-distal driving and post-treatment cephalometric radiographs and tracings, and Table 1 shows the respective values.

**Figure 8:**
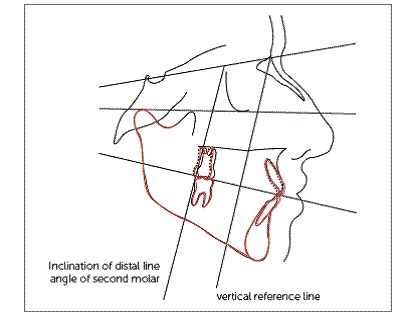
Pre- (black line) and post- (dash line) distal driving and post-treatment (red line) lateral cephalometry superimpositions on S-Na.

**Table 1:** Cephalometric comparisons before and after distal driving and post-treatment.

	Pre distal driving	Post distal driving	Post-treatment
SN-FH (degrees)	11	11	11
SNA (degrees)	83.5	83.5	83.5
SNB (degrees)	79.5	79.0	79.0
FMA (degrees)	27.5	27.8	27.7
U1 to FH (degrees)	110.5	108	113
Inclination of distal line angle of second molar to SN (degrees)	64.5	65.3	65
Mini-screw Axis to SN (degrees)	52.2	49.8	NA
Na-Menton distance (mm)	11.4	11.4	11.3
Distal cusp tip of first molar to VR* (mm)	16.9	20.4	20.2

*VR: vertical reference line (perpendicular line to occlusal plane from Na point).

To measure molar distalization, the most occlusal point on the distal cusp of the first molar was located, and its distance to a perpendicular line drawn from Na to the occlusal plane, used as a vertical reference, was assessed (Fig 8).

The changes of angle between the distal line angle of second molar and SN were assessed as molar inclination changes. Changes between the mandibular plane angle and Frankfort plane as well as Na-Menton distance were measured as vertical changes (Table 1).

**Figure 9:**
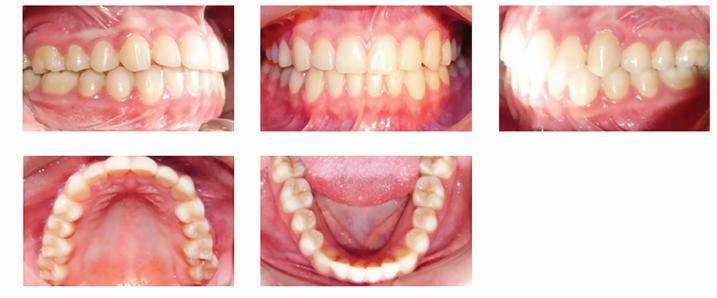
16-month follow-up after distal driving.

The 16-month follow-up after distal driving is seen in Figure 9.

## DISCUSSION

In this case report, we evaluated the clinical effectiveness of bone-anchored mini-screw-supported modified Hyrax appliance presented for bodily and *en-masse* bilateral posterior teeth distalization.

In before-used distalizing methods, anchorage preparations were extraoral anchorage;^2^ occlusal wire rests; palatal acrylic button, for instance, pendulum appliance; skeletal anchorage, such as combination of palatal acrylic button with mini-screw;^30^ and the zygoma anchorage system for buccal segment distalization.^34^ ^,^ ^35^ ^,^ ^36^

In distalization appliances, which use first or second premolars for anchorage, reaction forces lead to mesial crown tip of premolars and canines, and finally proclination of incisors.^41^

Extraoral appliances, such as headgear, have no reaction on anterior teeth, but success relies on patient's compliance. Distal crown tipping, extrusion and distal rotation of molars may occur as well. In addition, the force that is applied to patient's neck with the headgear produces a non physiological strain on neck muscles and the cervical spine.^33^

Palatal acrylic button used as anchorage hinders proper oral hygiene. It also applies reactive forces and moments to anterior teeth, and has some contraindications regarding dentition stages and local anatomy.^26^

In bone-anchored devices (osseointegrated implants, titanium mini-screws and mini-plates), most of these complications are solved. The advantages of mini-screws are as follows: no need for osseointegration, more application sites, as well as simple and less aggressive insertion and removal processes.^29^ Many investigations have used them to distalize one molar on each side of the maxilla, but we used mini-screws for bilateral *en-masse* distalization of all posterior teeth. We used mini-screws in para-median of anterior palate, with better bone density and thickness relative to buccal cortices. This site does not interfere in root movement, thus eliminating the need for mini-screw transposition during distal driving. This is another advantage of the presented method in comparison to previous ones.

Kaya et al used the zygoma anchorage system to distalize maxillary premolars and molars simultaneously.^34^ Limitations of zygoma-gear appliance are as follows: aggressive insertion and removal surgical procedures, facial inflammation for a number of days after surgery and the possibility of infection.^36^

Backward rotation of the mandible is not usually favorable during distalization; therefore, trying to achieve bodily movement of molars with minimal rotation and distal crown tipping, in addition to suitable case selection according to growth pattern, is important.^42^ Burhan controlled most of these unfavorable changes by night time application of high-pull headgear along with the frog appliance.^43^

For bodily movement, the vector of distalizing force should pass through the center of resistance of the target segments, e.g., heavy rods (power arms) should be used to control the direction of force.^25^ With the Frog appliance,^25^ the Distal Jet,^16^ ^,^ ^17^ ^,^ ^18^the Keles slider,^19^ Zygoma-Gear Appliance^36^ and Miniscrew Implant Supported Distalization System (MISDS),^33^ ^,^ ^44^ the force vector is approximately at the level of the center of resistance of the first molar. The higher vertical position of the hook on mesially extended transpalatal bar and MI-supported S-sheath makes the line of action of force higher than the center of resistance of the molar segment to set distalizing and intruding molars.^38^

In this study, the appliance was positioned near the palatal vault, 13 mm apical to the occlusal surface of maxillary molars. The screw was activated once a week, and produced 3.5-mm bodily distal movement of all posterior teeth simultaneously.

The results of a review^45^ revealed that the mean distal movement of maxillary molars was 0.7 mm per month (range of 0.2-1.2 mm). The slowest rate observed was with the Skeletal Anchorage System (SAS),^35^ ^,^ ^46^ and the fastest was seen for the Dual-Force Distalizer.^37^ Furthermore, it is likely that comparable overall treatment results can be achieved faster with the SAS rather than with the dual-force distalizer.^45^ In our study, the rate of *en-masse* distalization was 0.7 mm per month and faster than *en-masse* distalization with the SAS system.

The advantages of the method presented in this paper are predictability, good esthetics, immediate force application, bodily *en-masse* distalization without rotation and tipping of posterior teeth, easily insertion and removal of appliance. The patient did not report any significant pain or discomfort during Hyrax activations. 

The appliance can remain until anterior retraction completion as anchorage reinforcement, reducing concerns about relapse. Distalization mostly relapsed through fixed orthodontic therapy, but did not show any significant change in the post-retention period.^47^ Attachment of the appliance at two points in the anterior palate can resist against possible rotational movements of the appliance due to uneven distalization.

The suggestive indications for this mini-screw-supported modified hyrax appliance include: Class II molar relationship, distalization of maxillary posterior teeth in dental maxillary protrusion patients with previous extraction or congenital missing of maxillary premolars, and to provide space for decompensation in pre-surgical orthodontics for severe Class III orthognathic surgical cases with previous extraction of maxillary premolars.^35^

The probable disadvantages of this method include the need for patient's compliance for accurate oral hygiene and screw activation, slight pain during palatal anesthesia (relative to non skeletal anchorage methods), possibility of impingement of appliance components to palatal tissues due to loosening of mini-screws. Nevertheless, the patient reported herein did not have any of them. Previous third molar extraction before molar distalization is another disadvantage of this method.

## CONCLUSIONS

The novel method with mini-screw-supported modified Hyrax appliance presented in this study might be used for bodily, bilateral and *en-masse* distalization of maxillary posterior teeth without any unwanted movements of anterior teeth. This can reduce treatment duration and expand the orthodontist's potential to provide space and anchorage.
